# TEM-Net: A Tri-Channel Edge-Aware Multi-Scale Network for Thyroid Nodule Segmentation in Ultrasound Images

**DOI:** 10.3390/bioengineering13070810

**Published:** 2026-07-15

**Authors:** Yifei Peng, Zeru Hai, Feng Dong, Bisheng Tang, Yaoqun Wu, Xiaoyan Kui, Beiji Zou

**Affiliations:** 1 School of Information Science and Engineering, Shaoyang University, Shaoyang 422000, China; yfpeng6909@126.com (Y.P.); dongfeng@hnsyu.edu.cn (F.D.); ucas459@gmail.com (B.T.); wuyaoqun@mail.hnsyu.edu.cn (Y.W.); bjzou@csu.edu.cn (B.Z.); 2 Provincial Key Laboratory of Informational Service for Rural Area of Southwestern Hunan, Shaoyang University, Shaoyang 422000, China; 3 School of Computer Science and Engineering, Central South University, Changsha 410083, China

**Keywords:** thyroid nodule segmentation, ultrasound image segmentation, tri-channel contrast–gradient representation, edge-guided feature amplification, cross-scale attention refinement, deep learning

## Abstract

With the increasing detection of thyroid nodules in ultrasound screening, accurate nodule segmentation has become important for computer-aided assessment of clinically relevant features, such as contour regularity, aspect ratio, and margin sharpness. Although ultrasound is widely used as a first-line imaging modality in clinical practice, thyroid nodule segmentation remains challenging because of low tissue contrast, speckle-blurred boundaries, and large variations in nodule size and morphology. To address these challenges, we propose TEM-Net, a Tri-Channel Edge-Aware Multi-Scale Network for thyroid nodule segmentation. TEM-Net constructs a tri-channel representation from the raw ultrasound image, including the original grayscale image, a contrast-enhanced image, and a gradient-magnitude map, to highlight weak intensity differences and boundary-related cues in low-contrast ultrasound images. An Edge-Guided Feature Amplification (EGFA) module is introduced before the first down-sampling operation to emphasize boundary responses before spatial resolution is reduced. In addition, a Multi-Focus Cross-Scale Attention Refinement (MF-CAR) module is embedded into skip connections, combining dilated depth-wise convolutions with channel–spatial attention to improve the fusion of local boundary details and broader contextual information. Across three random seeds, TEM-Net achieves mean Dice scores of 0.8822 and 0.9066 and mean IoU scores of 0.7893 and 0.8291 on TN3K and DDTI, respectively, showing competitive performance compared with representative segmentation methods.

## 1. Introduction

Thyroid nodules are frequently detected during ultrasound (US) screening, with previous studies reporting that approximately 50–66% of adults may have at least one thyroid nodule [1]. Although only a small proportion of thyroid nodules are malignant, timely identification of suspicious nodules remains important because advanced thyroid cancer may be associated with extrathyroidal extension or distant metastasis [2]. Ultrasound is widely used as a first-line imaging modality for thyroid nodule assessment because it is non-invasive, cost-effective, widely available, and free of ionizing radiation [3,4]. Clinical guidelines, including the ACR TI-RADS, incorporate margin characteristics as important criteria for malignancy risk stratification [5,6]. For example, lobulated or irregular margins can increase the TI-RADS score and may influence follow-up or biopsy recommendations. Boundary-related characteristics are relevant to thyroid nodule risk stratification and may influence subsequent clinical assessment [7].

In clinical and research settings, visual assessment and manual delineation of thyroid nodules in grayscale ultrasound images remain operator-dependent and may be subject to inter-observer variability [8,9]. This process is particularly challenging when nodule boundaries are affected by speckle noise, low contrast, or ambiguous surrounding structures [10]. Automated thyroid nodule segmentation may therefore support quantitative measurement, follow-up comparison, and computer-aided biomedical image analysis [11,12].

Deep learning-based segmentation networks, such as U-Net and its variants, have been widely investigated for medical image segmentation and thyroid ultrasound analysis [13,14,15,16]. Further developments, such as the DeepLab series, improve receptive-field coverage by using dilated convolutions and atrous spatial pyramid pooling (ASPP) [17]. Transformer-based architectures, such as Swin-UNet [18] and TransUNet [19], have also been explored to model long-range dependencies in segmentation tasks.

**Figure 1:**
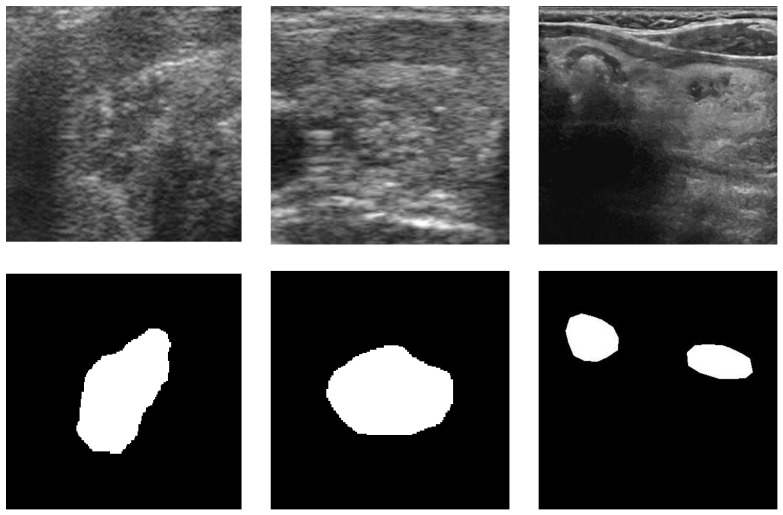
Challenging thyroid ultrasound images and corresponding annotations from the TN3K dataset, illustrating typical difficulties such as low contrast, blurred boundaries, and multiple nodules. In the annotation masks, white regions denote thyroid nodules and black regions denote background.

Despite these advances, automated thyroid nodule segmentation remains difficult in the presence of several ultrasound-specific image characteristics, as illustrated in Figure 1. First, thyroid nodules often exhibit low contrast against surrounding parenchyma, making intensity-based discrimination difficult for segmentation networks. Second, nodule boundaries can be indistinct or affected by speckle noise, and irregular margins may gradually blend into adjacent tissue, reducing the reliability of edge cues. Third, thyroid nodules vary substantially in size and morphology, ranging from small lesions to large or multifocal nodules with irregular shapes. Such scale and shape diversity makes it difficult for networks with limited receptive-field adaptability to capture fine boundary details and broader contextual information simultaneously.

To address these challenges, we develop TEM-Net and make the following contributions:

**(1) Tri-Channel Contrast–Gradient Fusion (TCGF):** We construct a three-channel representation from the raw B-mode image, its contrast-enhanced version, and a gradient-based edge map. This representation is designed to expose weak intensity differences and boundary-related cues in low-contrast thyroid ultrasound images.

**(2) Edge-Guided Feature Amplification (EGFA):** We introduce a Sobel-based gating mechanism before the first down-sampling layer to emphasize boundary responses before spatial resolution is reduced.

**(3) Multi-Focus Cross-Scale Attention Refinement (MF-CAR):** We embed parallel dilated depth-wise convolutions and channel–spatial attention into skip connections. This design aims to improve the fusion of local boundary details and broader contextual information for nodules with different sizes and shapes.

## 2. Related Work

Early studies on thyroid nodule analysis in ultrasound images commonly used handcrafted texture features, such as gray-level co-occurrence matrices (GLCM), local binary patterns (LBP), and Gabor filters, together with shallow classifiers or boundary-based models, including support vector machines (SVM), k-nearest neighbors (KNN), and active contours [20,21,22]. For example, several methods extracted statistical descriptors, such as energy, contrast, and entropy, from GLCM features and used SVM-based models for thyroid nodule classification [23]. However, handcrafted-feature-based models can be sensitive to speckle noise and scanner-dependent gain variations, which may reduce their robustness across different ultrasound devices [24]. Moreover, traditional pipelines usually separate feature extraction from classification or segmentation, limiting end-to-end optimization. This separation may reduce adaptability under conditions of poor boundary definition or low signal intensity [25,26,27].

Convolutional encoder–decoder networks have promoted the use of deep learning for thyroid nodule segmentation and have improved feature learning compared with handcrafted pipelines [28]. Existing deep learning approaches can be broadly grouped according to their architectural designs. U-Net [29] and its variants, including U-Net++ [30] and Attention U-Net [31], improve encoder–decoder feature fusion through dense skip connections and attention mechanisms. More recently, CE-UNet [32] introduced contrast-sensitive channel attention with sliding-window channel interaction to enhance thyroid nodule segmentation in ultrasound images. Although these models are effective in many medical segmentation tasks, their performance may be affected by thyroid ultrasound-specific factors, including low tissue contrast, speckle-obscured boundaries, and variations in nodule size and shape. To reduce computational cost, lightweight models such as DACNet [33] and DCSAU-Net [34] employ depthwise separable convolutions and compact feature-fusion modules. However, strong compression may limit the ability of some lightweight models to capture fine boundary details and multi-scale contextual information, which are important for delineating nodules with heterogeneous appearances.

Transformer-based architectures have been introduced to model long-range dependencies through self-attention, complementing the local receptive fields of convolutional networks. Hybrid frameworks, including Enhanced-TransUNet [35] and UCTransNet [36], integrate Transformer-based modules into U-Net-like backbones to combine local features with broader contextual representations. Recent methods such as MLMSeg [37] further explore scale-adaptive segmentation by combining convolutional features, Transformer-based contextual modeling, and graph-based structural information. However, self-attention-based designs can introduce considerable computational and memory costs, which may restrict their use in resource-constrained settings. In addition, their performance under ultrasound-specific artifacts, such as speckle noise and ambiguous margins, still requires further evaluation on thyroid-focused datasets. More recently, state space models (SSMs), such as VM-UNet [38] and UltraLight VM-UNet [39], have been explored as efficient alternatives for medical image segmentation. These architectures are designed to reduce sequence-modeling complexity while maintaining long-range modeling capability. Despite their efficiency, their behavior under common ultrasound imaging challenges, including noise interference, acoustic shadowing, and ambiguous boundaries, remains worth further investigation in complex thyroid nodule cases.

Motivated by these challenges, we propose TEM-Net, a tri-channel edge-aware multi-scale network for thyroid nodule segmentation. TEM-Net constructs a three-channel representation from the raw grayscale image, its contrast-enhanced version, and a gradient-magnitude map to expose intensity, contrast, and boundary-related cues. This contrast–gradient representation is intended to enrich early visual cues for low-contrast regions at the input stage. An edge-guided feature amplification module is applied before the initial down-sampling operation to emphasize boundary responses in regions with weak or indistinct contours. Furthermore, a Multi-Focus Cross-Scale Attention Refinement module is integrated into skip connections to improve the fusion of local boundary details and broader contextual information. By combining contrast–gradient input representation, early edge-guided feature amplification, and cross-scale skip refinement, TEM-Net provides a focused framework for thyroid nodule segmentation in ultrasound images.

## 3. Method

**Figure 2:**
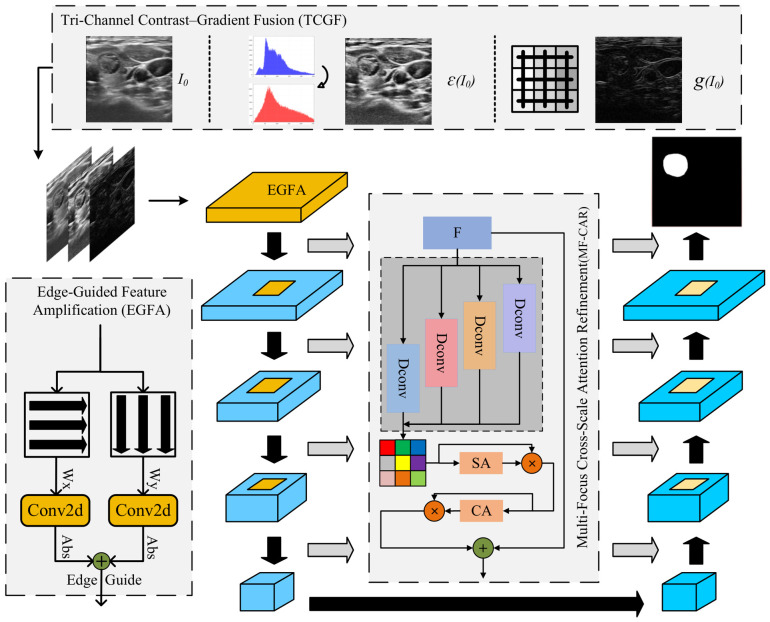
Overall architecture of TEM-Net. The symbol “×” denotes element-wise multiplication, and “+” denotes element-wise addition.

The overall architecture of TEM-Net is shown in Figure 2. Given an ultrasound frame $I_{0}\in R^{H\times W}$, we first construct a three-channel tensor $X={\left[I_{0},\;\varepsilon \left(I_{0}\right),\;g\left(I_{0}\right)\right]}^{T}\in R^{3\times H\times W}$, where ε(·) denotes tile-wise contrast enhancement, and g(·) denotes the normalized gradient magnitude. This TCGF representation provides the network with raw intensity, locally enhanced contrast, and gradient-based edge information while keeping the backbone architecture unchanged.

The tensor *X* is then processed by an Edge-Guided Feature Amplification (EGFA) module, which produces an edge-aware representation $\tilde{X}=X+X⊙M$, where $M\in {\left[0,1\right]}^{3\times H\times W}$ is generated from a fixed gradient map through a 1×1 convolution followed by a sigmoid function. EGFA introduces an explicit edge-guided modulation before the first resolution reduction.

The backbone follows a U-Net-like four-stage convolutional encoder–decoder architecture, and its detailed stage-wise configuration is provided in Table 1. Encoder features are passed to the decoder through skip connections, denoted as $\left\{S_{k}\right\}$. Each skip feature $S_{k}$ is refined by a Multi-Focus Cross-Scale Attention Refinement (MF-CAR) block, formulated as ${\hat{S}}_{k}=S_{k}+\psi \left(S_{k}\right)$, where ψ(·) aggregates multi-dilated depth-wise responses, applies channel re-scaling, and performs spatial gating with a large-kernel mask. This operation is designed to improve the fusion of local boundary details and broader contextual information, which may help represent small or spatially separated nodules.

The decoder uses bilinear up-sampling and double-convolution blocks to generate the final probability map $\hat{Y}$. The network is trained with a compound loss, $L=\mathrm{BCE}\left(\hat{Y},Y\right)+\mathrm{Dice}\left(\hat{Y},Y\right)$, which combines pixel-wise classification supervision with region-level overlap optimization. Overall, TCGF enriches the input representation with contrast and gradient cues, EGFA emphasizes early boundary responses, and MF-CAR refines skip features across different receptive fields. Together, these components form an edge-aware and cross-scale segmentation framework for thyroid ultrasound images.

### 3.1. Tri-Channel Contrast–Gradient Fusion (TCGF) Input Representation

To improve the representation of low-contrast thyroid ultrasound images, we construct a three-channel tensor from the original B-mode frame and provide it to the encoder as input. Let $I_{0}:\Omega \subset R^{2}\to \left[0,255\right]$ denote the original B-mode frame, where x∈Ω represents a pixel location. The first channel is the raw ultrasound image, defined as

(1) $$I_{\mathrm{raw}}\left(x\right)=I_{0}\left(x\right),$$

which preserves the original echogenic information.

For the second channel, we apply tile-wise contrast-limited adaptive histogram equalization $\varepsilon_{\alpha ,T}$, with clip limit α=2.0 and tile size T=8, to locally adjust the gray-level distribution and enhance low-contrast regions:

(2) $$I_{\mathrm{enh}}\left(x\right)=\varepsilon_{\alpha ,T}\;\left[I_{0}\right]\left(x\right).$$

The third channel captures gradient-based boundary information. Spatial derivatives are computed using first-order finite differences, and their Euclidean norm is normalized to the image intensity range:

(3) $$G\left(x\right)=\sqrt{{\left(\partial_{x}I_{0}\right)}^{2}+{\left(\partial_{y}I_{0}\right)}^{2}}.$$

(4) $$I_{\mathrm{grad}}\left(x\right)=255\cdot \frac{G\left(x\right)-\min_{\Omega }G}{\max_{\Omega }G-\min_{\Omega }G+\delta },$$

where $\delta =10^{-8}$ is a small constant used for numerical stability.

Unlike the learnable edge-guided module introduced later, $I_{\mathrm{grad}}$ is a fixed gradient-derived map that provides explicit boundary-related cues at the input stage. The final three-channel representation is defined as the ordered stack

(5) $$X\left(x\right)={\left[I_{\mathrm{raw}}\left(x\right),\;I_{\mathrm{enh}}\left(x\right),\;I_{\mathrm{grad}}\left(x\right)\right]}^{⊤}\in R^{3}.$$

By combining the raw image, the contrast-enhanced image, and the normalized gradient map, the TCGF representation provides the first convolutional layer with intensity, local-contrast, and boundary-related information without introducing additional trainable parameters.

### 3.2. Edge-Guided Feature Amplification (EGFA)

**Figure 3:**
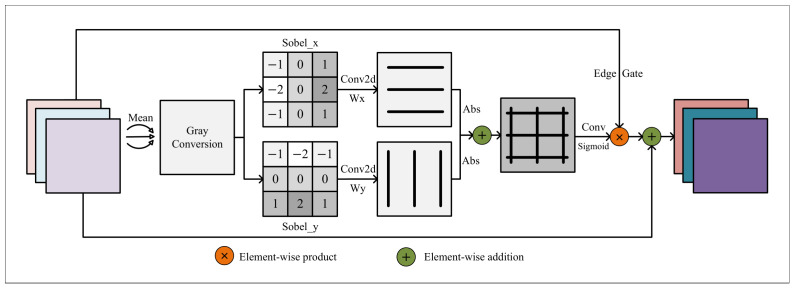
Structure of the Edge-Guided Feature Amplification (EGFA) module.

The Edge-Guided Feature Amplification (EGFA) module is inserted before the initial down-sampling operation to emphasize boundary-related responses at the early encoding stage, as shown in Figure 3.

Given the input tensor $X\in R^{B\times 3\times H\times W}$, a grayscale map is obtained by channel averaging:

(6) $$I_{\mathrm{gray}}=\frac{1}{3}\sum_{c=1}^{3}X_{c}.$$

Horizontal and vertical gradients are extracted using fixed Sobel kernels $W_{x}$ and $W_{y}$:

(7) $$W_{x}=\left[\begin{array}{ccc} -1 & 0 & 1\\ -2 & 0 & 2\\ -1 & 0 & 1 \end{array}\right],\;W_{y}=\left[\begin{array}{ccc} -1 & -2 & -1\\ \;\;0 & \;\;0 & \;\;0\\ \;\;1 & \;\;2 & \;\;1 \end{array}\right].$$

The corresponding gradient responses are computed as

(8) $$G_{x}=I_{\mathrm{gray}}*W_{x},\;G_{y}=I_{\mathrm{gray}}*W_{y},$$

where ∗ denotes two-dimensional convolution. An edge-response map is then computed as

(9) $$E=|G_{x}\left|+\right|G_{y}|.$$

To transform this single-channel edge response into a three-channel modulation mask, *E* is passed through a 1×1 convolution followed by a sigmoid function:

(10) $$M=\sigma \;\left(W_{e}*E\right),\;W_{e}\in R^{3\times 1\times 1\times 1},\;M\in {\left(0,1\right)}^{B\times 3\times H\times W},$$

where $W_{e}$ denotes a learnable 1×1 convolution kernel that directly projects the single-channel edge response $E\in R^{B\times 1\times H\times W}$ to a three-channel modulation mask. No intermediate channel is used in this projection.

The output of EGFA is defined as a residual edge-modulated representation:

(11) $$X_{\mathrm{egfa}}=X+X⊙M=X\left(1+M\right),$$

where ⊙ denotes the Hadamard product. Equation (11) increases features according to the learned edge-response mask, allowing boundary-related regions to receive larger weights while retaining the original residual information.

EGFA introduces only a small number of additional parameters and limited computational overhead because it uses fixed Sobel kernels and a lightweight 1×1 projection. This design incorporates explicit Sobel-based edge information into early feature modulation, which is intended to improve the representation of weak or blurred boundaries in thyroid ultrasound images.

### 3.3. Multi-Focus Cross-Scale Attention Refinement (MF-CAR) Block

**Figure 4:**
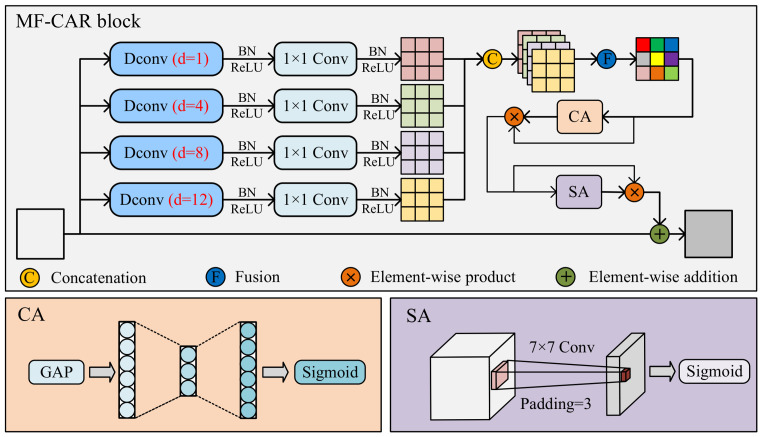
Structure of the Multi-Focus Cross-Scale Attention Refinement (MF-CAR) block.

To improve multi-scale feature refinement before decoder fusion, we apply Multi-Focus Cross-Scale Attention Refinement (MF-CAR) blocks to the deeper encoder features and the bottleneck feature. As illustrated in Figure 4, MF-CAR uses parallel dilated depth-wise convolutions followed by branch-specific point-wise projections, feature fusion, and sequential channel–spatial attention to recalibrate feature responses.

Let $F\in R^{B\times C\times H\times W}$ denote an input feature tensor. Given a dilation set D={1,4,8,12} with K=4, four branches are applied in parallel. Each branch consists of a 3×3 dilated depth-wise convolution followed by batch normalization and ReLU activation, and then a branch-specific 1×1 point-wise convolution with batch normalization and ReLU activation:

(12) $$F_{d_{k}}=\rho \left(\mathrm{BN}\left(P_{1\times 1}^{\left(k\right)}\left(\rho \left(\mathrm{BN}\left({\mathrm{DConv}}_{3\times 3}^{\left(d_{k}\right)}\left(F\right)\right)\right)\right)\right)\right),\;d_{k}\in D,$$

where ρ(·) denotes the ReLU activation, ${\mathrm{DConv}}_{3\times 3}^{\left(d_{k}\right)}$ denotes a 3×3 depth-wise convolution with dilation rate $d_{k}$ and padding $d_{k}$, and $P_{1\times 1}^{\left(k\right)}$ denotes the branch-specific 1×1 point-wise convolution. In our implementation, the four dilation rates are $d_{1}=1$, $d_{2}=4$, $d_{3}=8$, and $d_{4}=12$.

The branch outputs are concatenated along the channel dimension and further fused by a 1×1 convolution followed by batch normalization and ReLU activation:

(13) $$\tilde{F}=\rho \left(\mathrm{BN}\left(W_{f}*\left[F_{d_{1}}\;∥\;\cdots \;∥\;F_{d_{K}}\right]\right)\right),$$

where ∥ denotes channel-wise concatenation, and $W_{f}\in R^{C\times (KC)\times 1\times 1}$ denotes the 1×1 fusion convolution kernel.

Global average pooling is then applied to produce a channel descriptor $z\in R^{B\times C}$:

(14) $$z_{b,c}=\frac{1}{HW}\sum_{i=1}^{H}\sum_{j=1}^{W}{\tilde{F}}_{b,c,i,j}.$$

The channel attention branch computes channel weights as follows:

(15) $$w_{\mathrm{ch}}=\sigma \left(W_{2}\rho \left(W_{1}z\right)\right),\;W_{1}\in R^{\frac{C}{r}\times C},\;W_{2}\in R^{C\times \frac{C}{r}},$$

where σ(·) denotes the sigmoid function, and r=8 is the reduction ratio. The channel-refined feature is then obtained as

(16) $$F_{\mathrm{ch}}=\tilde{F}⊙w_{\mathrm{ch}},$$

where ⊙ denotes element-wise multiplication.

A spatial attention map is generated directly from the channel-refined feature using a 7×7 convolution followed by a sigmoid function:

(17) $$w_{\mathrm{sp}}=\sigma \left({\mathrm{Conv}}_{7\times 7}\left(F_{\mathrm{ch}}\right)\right),\;w_{\mathrm{sp}}\in {\left(0,1\right)}^{B\times 1\times H\times W}.$$

Finally, the spatially reweighted feature is combined with the original input through residual addition:

(18) $$F_{\mathrm{MF-CAR}}=F+F_{\mathrm{ch}}⊙w_{\mathrm{sp}}.$$

### 3.4. Loss Function

To train the segmentation network with both pixel-wise and region-level supervision, we use a compound loss consisting of binary cross-entropy (BCE) loss and Dice loss. The BCE term provides pixel-wise classification supervision, whereas the Dice term helps account for foreground–background imbalance by directly optimizing region overlap.

Let $p\in {\left[0,1\right]}^{N}$ denote the flattened predicted probability map, and let $y\in {\left\{0,1\right\}}^{N}$ denote the corresponding binary ground-truth mask. The total loss is defined as

(19) $$L_{\mathrm{total}}=\lambda_{\mathrm{BCE}}L_{\mathrm{BCE}}+\lambda_{\mathrm{Dice}}L_{\mathrm{Dice}}.$$

where $\lambda_{\mathrm{BCE}}$ and $\lambda_{\mathrm{Dice}}$ are fixed weighting coefficients, both set to 1.0 in our implementation.

The BCE loss is defined as

(20) $$L_{\mathrm{BCE}}=-\frac{1}{N}\sum_{i=1}^{N}\left[y_{i}\log\left(p_{i}\right)+\left(1-y_{i}\right)\log\left(1-p_{i}\right)\right].$$

The Dice loss is expressed as

(21) $$L_{\mathrm{Dice}}=1-\frac{2\sum_{i=1}^{N}p_{i}y_{i}+\varepsilon }{\sum_{i=1}^{N}p_{i}+\sum_{i=1}^{N}y_{i}+\varepsilon },$$

where $\varepsilon =10^{-6}$ is used for numerical stability.

This combination jointly considers pixel-wise prediction and region-level overlap, which is useful for thyroid ultrasound images because nodules may occupy a relatively small area and may have weak or blurred boundaries.

## 4. Experiments

### 4.1. Datasets

To evaluate the proposed method, we conducted experiments on two publicly available thyroid nodule segmentation datasets: TN3K and DDTI.

The TN3K dataset [40], curated by Gong et al., contains 3493 ultrasound images with pixel-level annotations and was acquired at Zhujiang Hospital of Southern Medical University under ethical approval. Following the commonly used evaluation protocol for TN3K, 614 images were reserved for testing. The remaining 2879 images were used as the training pool, from which 20% (576 images) were randomly selected for validation, leaving 2303 images for training.

The DDTI dataset [41], developed by Pedraza et al., contains 637 ultrasound images with thyroid nodule annotations. For this dataset, we used a random stratified split with a ratio of 7:1:2, resulting in 445 training images, 63 validation images, and 129 testing images.

All images and corresponding segmentation masks were resized to 224×224 pixels to keep the input size consistent during training and evaluation. Comparative experiments were conducted on both TN3K and DDTI, while ablation experiments were performed on TN3K to examine the contribution of each module.

### 4.2. Implementation Details

All experiments were implemented in PyTorch 1.13 and conducted on a workstation equipped with a single NVIDIA RTX A6000 GPU with 48 GB of memory. TEM-Net was trained for 300 epochs with a batch size of 32 using the AdamW optimizer [42], with an initial learning rate of $1\times 10^{-3}$. A cosine annealing learning-rate scheduler [43] was used to update the learning rate during training.

Data augmentation was applied during training to reduce overfitting. The augmentation operations included random horizontal flipping, random vertical flipping, and random rotation.

### 4.3. Evaluation Metrics

To evaluate segmentation performance, we used five commonly adopted metrics: Intersection over Union (IoU), Dice coefficient, accuracy (ACC), specificity (Spe), and sensitivity (Sen). These metrics evaluate region overlap and pixel-wise classification performance from different perspectives. The metrics are computed as follows:

(22) $$\mathrm{IoU}=\frac{TP}{TP+FP+FN}.$$

(23) $$\mathrm{Dice}=\frac{2\cdot TP}{2\cdot TP+FP+FN}.$$

(24) $$\mathrm{Accuracy}=\frac{TP+TN}{TP+TN+FP+FN}.$$

(25) $$\mathrm{Specificity}=\frac{TN}{TN+FP}.$$

(26) $$\mathrm{Sensitivity}=\frac{TP}{TP+FN}.$$

where TP, FP, TN, and FN denote true positives, false positives, true negatives, and false negatives, respectively. In addition, we report the number of parameters (Params, M) and the computational complexity measured by floating-point operations (GFLOPs). Statistical significance was assessed using a one-sided paired Wilcoxon signed-rank test based on seed-averaged per-image Dice scores. Specifically, for each test image, the Dice scores obtained from three random seeds were first averaged for each method. The paired Wilcoxon test was then performed between TEM-Net and each compared baseline, with the alternative hypothesis that TEM-Net achieves higher Dice scores. A *p*-value below 0.05 was considered statistically significant.

### 4.4. Comparison with Representative Segmentation Methods

We compared TEM-Net with eight representative segmentation methods, including CNN-based, Transformer-based, and Mamba-based models. For a fair comparison, all compared methods were trained for 300 epochs using the same data splits and evaluation protocol. When available, the default configurations from the original implementations were used. To improve reproducibility, the stage-wise backbone configuration of TEM-Net is summarized in Table 1, and the main configuration settings of the compared baseline methods are summarized in Table 2. Quantitative results on TN3K and DDTI are reported in Table 3 and Table 4, respectively.

U-Net++ [30] introduces nested and densely connected skip pathways to reduce the semantic gap between encoder and decoder features. DACNet [33] combines depth-wise separable convolutions, squeeze-and-excitation gating, and atrous dual-attention modules to reduce computational cost while preserving multi-scale contextual information. DCSAU-Net [34] uses compact split-attention blocks and asymmetric down-sampling to enhance deep–shallow feature fusion. CE-UNet introduces contrast-sensitive channel attention with sliding-window channel interaction for thyroid nodule segmentation [32].

TransUNet [19] embeds a Vision Transformer encoder into a U-Net-like architecture to model long-range dependencies. UCTransNet [36] replaces conventional skip connections with a Channel Transformer module for multi-scale channel-wise feature fusion. VM-UNet [38] adopts visual state-space blocks to model long-range interactions with reduced sequence-modeling complexity. Ultra-VM-UNet [39] is a compact variant of VM-UNet that introduces parallel Vision-Mamba layers with a small parameter budget.

As shown in Table 3, TEM-Net achieves the highest IoU, Dice, and accuracy among the compared methods on TN3K. Its specificity is comparable to the highest reported value. On DDTI, TEM-Net obtains the highest IoU, Dice, accuracy, and sensitivity. These results indicate strong overlap-based performance under the evaluated in-domain protocols.

**Figure 5:**
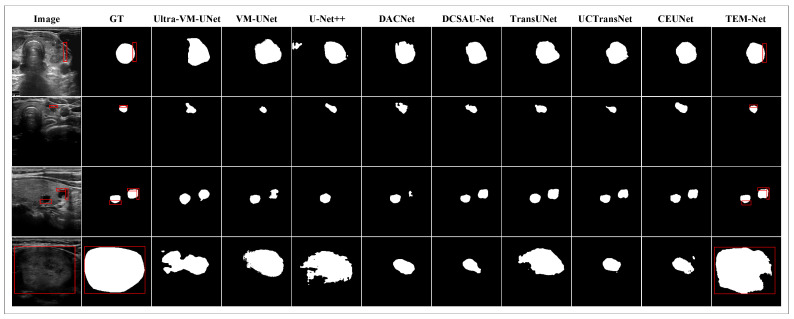
Qualitative comparison of segmentation results on the TN3K dataset. Red boxes indicate challenging regions with visible segmentation differences.

**Figure 6:**
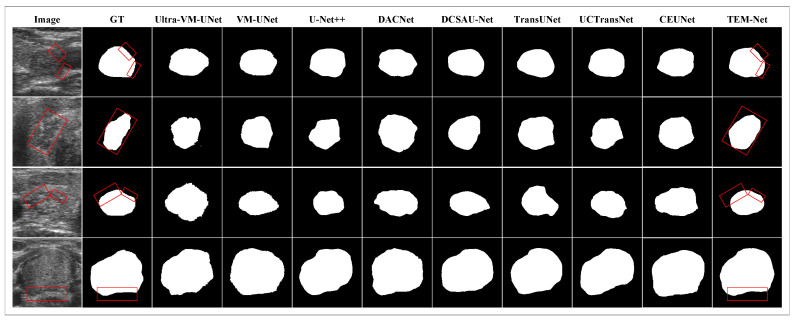
Qualitative comparison of segmentation results on the DDTI dataset. Red boxes indicate challenging regions with visible segmentation differences.

Figure 5 and Figure 6 show qualitative comparisons on TN3K and DDTI, respectively. The selected examples include challenging cases with weak boundaries, large nodules, small nodules, multiple nodules, and low contrast. In margin-challenging cases, TEM-Net maintains relatively coherent nodule-region predictions in the selected examples, whereas several compared methods exhibit local irregularities or leakage into adjacent tissue. This observation is consistent with the design motivation of EGFA, which emphasizes early boundary-related responses.

In examples containing small or spatially separated nodules, TEM-Net appears to maintain more coherent coverage of the visible nodule regions than several baseline methods. For large low-contrast lesions, TEM-Net shows fewer fragmented regions and more coherent overall region predictions. In low-contrast DDTI examples, the contrast-enhanced and gradient-derived channels may help highlight weak boundary-related cues. Overall, the qualitative results are generally consistent with the quantitative trends and suggest that TEM-Net can provide competitive segmentation performance for several challenging ultrasound image characteristics represented in examples.

### 4.5. Ablation Studies

This section presents ablation studies on the TN3K dataset to examine the contribution of each component in TEM-Net. All ablation experiments were conducted under a fixed random seed using the same TN3K split and training protocol.

**(1) Effect of TCGF, EGFA, and MF-CAR modules: **Table 5 shows the results obtained by progressively adding the proposed components to the baseline model. Compared with the baseline using a single-channel grayscale input, adding TCGF improves IoU from 0.7707 to 0.7770 and Dice from 0.8705 to 0.8745, corresponding to absolute improvements of 0.0063 and 0.0040, respectively. Sensitivity also increases from 0.8536 to 0.8596. These results suggest that the contrast-enhanced image and gradient-derived map provide useful additional cues for low-contrast thyroid ultrasound images.

Adding EGFA further improves IoU to 0.7822 and Dice to 0.8778. This result is consistent with the design motivation of EGFA, which emphasizes boundary-related responses before the first down-sampling operation. Finally, integrating MF-CAR into skip connections achieves the best IoU and Dice values in this ablation setting, reaching 0.7902 and 0.8828, respectively. Compared with the baseline, the full model improves IoU by 0.0195 and Dice by 0.0123. These results indicate that TCGF, EGFA, and MF-CAR provide cumulative improvements under the TN3K evaluation protocol.

**(2) Comparison of MF-CAR with existing attention and multi-scale modules: **Table 6 compares MF-CAR with representative attention and multi-scale modules. Compared with CBAM, MF-CAR improves IoU from 0.7843 to 0.7902 and Dice from 0.8791 to 0.8828. Sensitivity also increases from 0.8616 to 0.8732, while specificity decreases slightly from 0.9865 to 0.9856. This comparison indicates that the MF-CAR configuration is associated with higher foreground sensitivity under the evaluated setting.

Compared with Lite-ASPP, MF-CAR achieves slightly higher IoU (0.7902 vs. 0.7879) and Dice (0.8828 vs. 0.8813), with moderate increases in parameters and FLOPs. Compared with the full ASPP module, however, MF-CAR is marginally lower in IoU (0.7902 vs. 0.7906), Dice (0.8828 vs. 0.8831), accuracy (0.9720 vs. 0.9722), and specificity (0.9856 vs. 0.9862), while achieving slightly higher sensitivity (0.8732 vs. 0.8699). Thus, ASPP remains marginally better when overlap accuracy alone is prioritized.

Nevertheless, MF-CAR reduces the parameter count from 38.87 M to 18.54 M and FLOPs from 51.27 G to 29.52 G, corresponding to reductions of approximately 52.3% and 42.4%, respectively. Under the same experimental setting, the average epoch-level wall-clock time was 40.83 s for MF-CAR and 72.83 s for ASPP. Overall, these results indicate that MF-CAR provides a performance–efficiency trade-off rather than a uniform accuracy advantage over ASPP.

**(3) Effect of hybrid loss function:** Compared with BCE loss alone, adding Dice loss slightly improves the overlap-based metrics. As shown in Table 7, IoU increases from 0.7851 to 0.7902, Dice increases from 0.8796 to 0.8828, and sensitivity increases from 0.8676 to 0.8732. Specificity remains unchanged at 0.9856, while accuracy increases slightly from 0.9713 to 0.9720.

These results suggest that the Dice term helps account for foreground–background imbalance by directly optimizing region overlap. The improvement is modest but consistent across IoU, Dice, and sensitivity in this experiment. Therefore, the BCE+Dice loss is used as the default training objective in TEM-Net.

## 5. Discussion

On the two public thyroid nodule segmentation datasets used in this study, TN3K and DDTI, TEM-Net achieves the highest Dice and IoU scores among the compared methods. It uses 18.5 M parameters and 29.52 GFLOPs, indicating a moderate parameter count but a non-negligible computational cost compared with lightweight alternatives. Qualitative results further suggest that TEM-Net can produce relatively coherent nodule-region predictions in several challenging examples, such as weak-boundary nodules, low-contrast lesions, and small or multiple nodules.

These results are consistent with the design motivation of the three proposed modules. TCGF provides the first convolutional layer with raw-intensity, local-contrast, and gradient-derived boundary cues. EGFA emphasizes edge-related responses before the first resolution reduction. MF-CAR refines skip features through dilated depth-wise convolutions and channel–spatial attention, which may improve the fusion of local boundary details and broader contextual information. Together, these components provide an edge-aware and cross-scale framework for thyroid ultrasound image segmentation.

It should be noted that Dice and IoU quantify regional overlap between model predictions and reference annotations rather than direct clinical utility. This distinction is particularly relevant in thyroid ultrasound, where manual nodule delineation is observer-dependent [8,9]. Across three random seeds, TEM-Net achieved Dice/IoU values of 0.8822±0.0011/0.7893±0.0018 on TN3K and 0.9066±0.0010/0.8291±0.0016 on DDTI. The small variations across runs indicate stable overlap with the available reference annotations. These results support the potential use of TEM-Net as an automated segmentation component for region-level quantitative analysis, while multi-reader studies and direct comparison with inter-observer variability are still needed to establish clinical utility.

Several limitations warrant further investigation. First, the current pipeline processes only single B-mode ultrasound images. Incorporating additional ultrasound information, such as Doppler flow or elastography, may be useful for future studies involving more comprehensive thyroid nodule assessment [44]. Second, the local-contrast operator in TCGF uses fixed hyperparameters. Future work could investigate learnable or content-adaptive enhancement strategies to improve adaptability across imaging conditions. Third, cross-dataset transferability remains a clear limitation of the current study. The strong in-domain performance observed on TN3K and DDTI should not be interpreted as robustness to datasets with entirely unseen distributions. Future work should investigate dedicated domain adaptation and domain generalization strategies to further improve cross-dataset transferability. Fourth, although TEM-Net has a moderate number of parameters, its FLOP cost is higher than that of several lightweight models, and further model compression or inference-time analysis would be required for deployment-oriented applications. Finally, uncertainty estimation may help identify low-confidence predictions and support more cautious interpretation in ambiguous cases [45].

## 6. Conclusions

We introduced TEM-Net, a tri-channel edge-aware multi-scale network for thyroid nodule segmentation in ultrasound images. The method combines contrast–gradient input representation, early edge-guided feature amplification, and cross-scale skip-feature refinement. Experiments under the evaluated in-domain protocols on TN3K and DDTI show that TEM-Net achieves competitive Dice and IoU scores among the compared methods. These findings support TEM-Net as a competitive segmentation framework under the evaluated in-domain protocols, while broader cross-dataset generalizability remains to be established.

## Figures and Tables

**Table 1:** Backbone configuration of TEM-Net.

Stage	Resolution	Channels	Main Operation
Input	224×224	3	TCGF + EGFA
Stem	224×224	64	DC
Encoder 1	112×112	128	MP + DC
Encoder 2	56×56	256	MP + DC
Encoder 3	28×28	512	MP + DC
Bottleneck	14×14	512	MP + DC
Decoder 3	28×28	256	Up + Concat + DC
Decoder 2	56×56	128	Up + Concat + DC
Decoder 1	112×112	64	Up + Concat + DC
Decoder 0	224×224	64	Up + Concat + DC
Output	224×224	1	1×1 Conv + Sigmoid

Note: DC denotes two consecutive 3×3 convolution–batch normalization–ReLU units. MP denotes 2×2 max pooling, and Up denotes bilinear up-sampling. MF-CAR is applied to the deeper encoder features and the bottleneck feature before decoder fusion, while the shallow stem feature is directly connected to the final decoder stage to preserve low-level spatial details.

**Table 2:** Key configuration settings of the compared baseline models.

Architecture	Algorithm	Configuration Settings
CNN	U-Net++	filters: [32, 64, 128, 256, 512]
CNN	DACNet	filters: [32, 64, 128, 256, 512], split: fc, bridge: True
CNN	DCSAU-Net	block: Bottleneck, layers: [2, 2, 2, 2], bottleneck_width: 64, stem_width: 2
CNN	CE-UNet	channels: [64, 128, 256, 512, 1024], CEAM kernels: [3, 5]
Transformer	TransUNet	model: R50-ViT-B_16, activation: sigmoid, patch size: 16, token grid: 14×14
Transformer	UCTransNet	num_heads: 4, num_layers: 4, patch_sizes: [16, 8, 4, 2], expand_ratio: 4, KV_size: 960
Mamba	Ultra-VM-UNet	c_list: [8, 16, 24, 32, 48, 64], split_att: fc, bridge: True
Mamba	VM-UNet	depths: [2, 2, 2, 2], depths_decoder: [2, 2, 2, 1], drop_path_rate: 0.2

Note: All compared models were trained and evaluated using the same 224×224 input resolution. For TransUNet, the 14×14 token grid is obtained from the 224×224 input with a patch size of 16. For UCTransNet, patch sizes [16, 8, 4, 2] are applied to multi-scale encoder features, yielding aligned 14×14 token representations across scales.

**Table 3:** Quantitative comparison on the TN3K dataset with three random seeds.

Method	Params	FLOPs	IoU	Dice	Acc	Spe	Sen
Ultra-VM-UNet	0.05 M	0.05 G	0.6736±0.0168	0.8049±0.0119	0.9531±0.0036	0.9738±0.0041	0.8019±0.0107
VM-UNet	27.43 M	4.76 G	0.7335±0.0104	0.8462±0.0070	0.9684±0.0074	0.9796±0.0023	0.8424±0.0049
U-Net++	9.16 M	26.51 G	0.7714±0.0064	0.8710±0.0041	0.9694±0.0011	0.9851±0.0016	0.8554±0.0082
DACNet	1.28 M	0.56 G	0.7661±0.0050	0.8675±0.0032	0.9681±0.0008	0.9821±0.0013	0.8661±0.0066
DCSAU-Net	2.60 M	5.29 G	0.7781±0.0052	0.8752±0.0033	0.9699±0.0009	0.9833±0.0032	0.8733±0.0200
TransUNet	105.28 M	24.67 G	0.7798±0.0016	0.8763±0.0010	0.9702±0.0007	0.9832±0.0032	0.8753±0.0181
UCTransNet	66.43 M	32.98 G	0.7762±0.0037	0.8740±0.0023	0.9694±0.0007	0.9816±0.0022	0.8801±0.0117
CE-UNet	31.04 M	41.91 G	0.7757±0.0063	0.8737±0.0040	0.9697±0.0009	0.9837±0.0011	0.8675±0.0103
TEM-Net	18.54 M	29.52 G	0.7893±0.0018	0.8822±0.0011	0.9718±0.0001	0.9851±0.0015	0.8748±0.0105

Note: A one-sided paired Wilcoxon signed-rank test was conducted on seed-averaged per-image Dice scores between TEM-Net and each compared method. All *p*-values were below 0.05.

**Table 4:** Quantitative comparison on the DDTI dataset with three random seeds.

Method	Params	FLOPs	IoU	Dice	Acc	Spe	Sen
Ultra-VM-UNet	0.05 M	0.05 G	0.7827±0.0129	0.8781±0.0081	0.9296±0.0058	0.9398±0.0104	0.9035±0.0062
VM-UNet	27.43 M	4.76 G	0.8041±0.0144	0.8914±0.0088	0.9381±0.0050	0.9506±0.0045	0.9062±0.0108
U-Net++	9.16 M	26.51 G	0.8136±0.0015	0.8972±0.0009	0.9420±0.0005	0.9566±0.0056	0.9042±0.0131
DACNet	1.28 M	0.56 G	0.8086±0.0065	0.8942±0.0040	0.9400±0.0028	0.9542±0.0055	0.9037±0.0042
DCSAU-Net	2.60 M	5.29 G	0.8198±0.0003	0.9010±0.0002	0.9444±0.0012	0.9606±0.0094	0.9028±0.0201
TransUNet	105.28 M	24.67 G	0.8185±0.0042	0.9002±0.0026	0.9435±0.0014	0.9570±0.0041	0.9089±0.0101
UCTransNet	66.43 M	32.98 G	0.8213±0.0008	0.9019±0.0005	0.9447±0.0011	0.9574±0.0053	0.9120±0.0097
CE-UNet	31.04 M	41.91 G	0.8179±0.0082	0.8998±0.0049	0.9430±0.0029	0.9543±0.0063	0.9140±0.0131
TEM-Net	18.54 M	29.52 G	0.8291±0.0016	0.9066±0.0010	0.9471±0.0008	0.9597±0.0060	0.9149±0.0130

Note: A one-sided paired Wilcoxon signed-rank test was conducted on seed-averaged per-image Dice scores between TEM-Net and each compared method. All *p*-values were below 0.05.

**Table 5:** Ablation study of TEM-Net components on the TN3K dataset.

Model	IoU	Dice	Acc	Spe	Sen
Baseline	0.7707	0.8705	0.9693	0.9852	0.8536
Baseline + TCGF	0.7770	0.8745	0.9702	0.9854	0.8596
Baseline + TCGF + EGFA	0.7822	0.8778	0.9709	0.9851	0.8668
Baseline + TCGF + EGFA + MF-CAR	**0.7902**	**0.8828**	**0.9720**	**0.9856**	**0.8732**

**Table 6:** Comparison of MF-CAR with representative attention and multi-scale modules.

Module	Params	FLOPs	Epoch Time	IoU	Dice	Acc	Spe	Sen
CBAM	13.70 M	23.83 G	27.67 s	0.7843	0.8791	0.9714	0.9865	0.8616
Lite-ASPP	17.06 M	27.23 G	60.50 s	0.7879	0.8813	0.9718	0.9859	0.8690
ASPP	38.87 M	51.27 G	72.83 s	**0.7906**	**0.8831**	**0.9722**	0.9862	0.8699
MF-CAR	18.54 M	29.52 G	40.83 s	0.7902	0.8828	0.9720	0.9856	**0.8732**

**Table 7:** Comparison between BCE loss and the hybrid BCE + Dice loss.

Loss	IoU	Dice	Acc	Spe	Sen
BCE	0.7851	0.8796	0.9713	**0.9856**	0.8676
BCE + Dice	**0.7902**	**0.8828**	**0.9720**	**0.9856**	**0.8732**

Bold values indicate the best performance among the compared loss settings.

## Data Availability

The data analyzed in this study are publicly available thyroid ultrasound datasets. The TN3K and DDTI datasets can be obtained from their original dataset providers or corresponding publications. No new original dataset was generated in this study.
